# Rinsing with Saline Promotes Human Gingival Fibroblast Wound Healing *In Vitro*

**DOI:** 10.1371/journal.pone.0159843

**Published:** 2016-07-21

**Authors:** Nam Cong-Nhat Huynh, Vincent Everts, Chidchanok Leethanakul, Prasit Pavasant, Ruchanee Salingcarnboriboon Ampornaramveth

**Affiliations:** 1 Mineralized Tissue Research Unit, Faculty of Dentistry Chulalongkorn University, Bangkok, Thailand; 2 Department of Oral Cell Biology, Academic Centre for Dentistry Amsterdam (ACTA), University of Amsterdam and VU University Amsterdam, Research Institute MOVE, Gustav Mahlerlaan 3004, 1081 LA Amsterdam, The Netherlands; 3 Department of Preventive Dentistry, Faculty of Dentistry, Prince of Songkla University, Songkhla, Thailand; 4 Department of anatomy, Faculty of Dentistry Chulalongkorn University, Bangkok, Thailand; 5 Research Unit on Oral Microbiology and Immunology, Microbiology department, Faculty of Dentistry Chulalongkorn University, Bangkok, Thailand; University of Alabama at Birmingham, UNITED STATES

## Abstract

Rinsing the mouth with sodium chloride (NaCl) solution is believed to promote healthy gum and improve oral ulcer healing. Scientific evidence to support this assumption is, however, lacking. This study aims to investigate the effect and clarify underlying mechanisms of short-term rinsing with NaCl on human gingival fibroblast (hGFs) wound healing. Isolated primary hGFs and human normal oral keratinocytes (hNOKs) were rinsed with 0–7.2% NaCl for 2 min, 3 times a day. Scratch-tests, trans-well migration assays and MTT activity were performed. mRNA expression was assessed of type-I collagen, fibronectin and FAK. Changes in FAK and F-actin were detected by immunofluorescence. KCl, NaH_2_PO_4_, KH_2_PO_4_ were used to clarify the molecules involved. Rinsing with 0.9–1.8% NaCl significantly promoted hGFs cell migration but not proliferation. However, it had no effect on hNOKs. Rinsing with 1.8% NaCl significantly up-regulated the expression of type-I collagen and fibronectin. FAK and F-actin, molecules responsible for cytoskeleton re-organization and cell migration, were also up-regulated. Cl^-^ seemed to be essential since rinsing with KCl resulted in a similar effect as noted with NaCl. In conclusion, short-term rinsing with NaCl promoted hGFs migration, and increased the expression of extracellular matrix as well as cytoskeletal proteins. These data strongly support the long held belief in the benefits of using NaCl mouth-rinse.

## Introduction

Treating gum disease with saline rinses appeared in China as early as 2700 B.C. [1, 2]. It has long been believed that rinsing the mouth with sodium chloride (NaCl) solution can promote healthy gums and hasten oral ulcer healing. Nowadays, to maintain oral health, many dentists advise their patients to rinse their mouth with salt solution as supplementary to routine oral care. However, up to now, there is no scientific evidence proofing the efficiency of this simple method. There is also no recommended concentration of NaCl that is appropriate for oral rinse without causing tissue damage. The detailed mechanism of the effect of NaCl on gingival wound healing is still unknown.

During wound healing, tissue remodeling allows the replacement of the injured tissue. It has been hypothesized that fibroblasts play an active role. These cells are committed to repopulate damaged tissues through cell proliferation, differentiation, and migration. Among these process, cell migration is a critical biological response during wound-healing [3, 4]. Cell motility is stimulated by extracellular signals, which thereafter initiate intracellular signaling proteins that localize at the contact sites with the extracellular matrix, termed focal contacts. Focal adhesion kinase (FAK) is an intracellular protein-tyrosine kinase that regulates assembly and disassembly of focal contacts required for efficient movement of the cell [5].

Hyperosmolarity is a condition by which the osmolality of blood in our bodies is higher than 290 mOsmol/kg and the exposure of cells to a high salt concentration is a common phenomenon. Under different conditions or with certain cell types, hyperosmolarity can lead to cell damage or survival [6]. Several studies showed that exposure to appropriate hyperosmotic medium can increase cell diameter and adhesion area of fibroblasts and myoblasts [7, 8]. In comparison with skin fibroblasts, gingival fibroblasts express a specific phenotype in which molecules involved in regulation of inflammation and extracellular matrix remodeling were elevated [9, 10]. These inherent differences of the gingival fibroblast phenotype may underlie the ability of gingival wounds to heal faster with less scar formation as compared to skin wounds. Still there is no data available regarding the response of gingival fibroblast to a hyperosmolarity condition.

To investigate the effect of repeated short-term rinsing with NaCl solution on human gingival fibroblasts (hGFs) behavior, we hypothesize that NaCl can stimulate migration of primary hGFs and thereby *in vitro* wound healing. Migration will be analyzed in two systems, the scratch assay and a transwell system. In addition, some of the mechanisms underlying the effect of NaCl on gingival fibroblast behavior will also be elucidated.

## Materials and Methods

### Cell culture

Third molars from healthy young volunteers, age 18 to 25 year-old, were extracted as recommended by their dentist. Immediately after extraction, the teeth were transferred to the lab in ice cold storage medium DMEM (#11960, Gibco, Life Technologies Corporation, Grand Island, NY) supplemented with 10% FBS, 1% L-Gluamine, 0,5 mg/ml gentamicin and 3 mg/ml amphotericin B. The gingival tissues attached to the cervical area were carefully removed from the tooth and rinsed twice with PBS. The tissue was minced into 1x2 mm pieces with a surgical blade and seeded in DMEM supplemented with 10% FBS, 1% L-Gluamine, 1% antibiotics. The tissue samples were incubated at 37°C humidified atmosphere with 5% CO_2_, the medium was replaced every 3 days until outgrowing cells reached confluence. The primary hGFs at the 3^rd^–6^th^ passage were used for the experiments. The patients provided written informed consent for the use of discarded tissues for research purposes. Tissue samples were deidentified and analyzed anonymously. The Ethics Committee of the Faculty of Dentistry, Chulalongkorn University, Thailand has approved the study to be carried out according to the protocol and informed permission dated and/or amended as follows in compliance with the ICH/GCP (HREC-DCU 2015–068). Human normal oral keratinocytes (hNOKs) were cultured in keratinocyte-serum-free medium (#17005–042, Gibco) supplemented with Bovine Pituitary Extract (BPE) and human recombinant Epidermal Growth Factor (rEGF) (#13028–014, #10450–013, Gibco) as previous study [11]. The medium was changed every 5 days until 70% confluence for further experiments.

### Scratch-test assay (“wound healing assay”)

To evaluate the effect of NaCl on hGF and hNOK migration, the scratch-test assay was performed. Cells were seeded in 24-well-plate (39,500 cells/ cm^2^). After 24h, a sterile 200 μl pipette tip was used to make a straight scratch line on the monolayer of confluent cells at the bottom of the culture plate (simulating a wound). The debris was washed away with PBS and the cells were then cultured at 37°C, humidified 5% CO_2_. The cells were rinsed with 0, 0.9%, 1.8%, 3.6% or 7.2% NaCl in culture medium for 2 minutes, 3 times a day for 48h (every 5–6 hours). At time point 0, 24 and 48h the “wound” areas were observed and recorded by an inverted microscope and digital camera at the same position of culture plate. The area of “wound healing” was analyzed using the Image-Analysis J 1.45S software (Wayne Rasband, National Institutes of Health, Bethesda, MD) as described previously [12]. The remaining “wound” areas between groups were compared. With the same protocol 2.9% KCl, 10% NaH_2_PO_4_, 9.7% KH_2_PO_4_ were used to elucidate the candidate molecules that exerted the effect on the migration (the concentrations were calculated to be equivalent in molarity to NaCl, 0.121 M Na^+^ and 0.187 M Cl^-^).

### Cell proliferation assay

Cell viability was assessed by 3-(4,5-dimethylthiazol-2-yl)-2,5-diphenyl tetrazolium bromide assay (MTT, #298–931, USB Corporation, Cleveland, OH). The cells were plated at 2x10^4^ cells/cm^2^ in 24-well plate. After 24h, the medium was changed to serum-free medium for 4 hours and then un-scratched cells were rinsed with 0.9%, 1.8%, 3.6%, 7.2% NaCl or 1.8% NaCl, 2.9% KCl, 10% NaH_2_PO_4_, 9.7% KH_2_PO_4_ in culture medium for 2 minutes, 3 times a day. The control (untreated) group was rinsed with normal growth medium. After 48h, the medium was replaced with 0.5 ml MTT solution and incubated for 30 min at 37°C. The formazan product was dissolved in solubilization/stop solution. Using a microplate reader (ELx800, BioTek, Winooski, VT), the optical densities were measured at 570 nm. The difference of absorbance between groups presented the variety of cell numbers.

### Transwell migration assay

Cell migration assay was performed in 24-well size Transwell inserts with 8.0-μm-pore polycarbonate membrane and 0.3 cm^2^ effective growth area (#3422, BD FalconTM Cell Culture Inserts, BD Biosciences, Bedford, MA). hGFs were trypsinized and re-suspended in serum free media. 1x10^5^ cells in total 200 μl of media were seeded in each insert. One hour after seeding, the rinsing protocol was started. Briefly, the inserts were moved to new 24-well-plates contain 700 μl of 0, 0.9, 1.8, 3.6 or 7.2% NaCl or 1.8% NaCl, 2.9% KCl, 10% NaH_2_PO_4_, 9.7% KH_2_PO_4_ in serum free medium for 2 minutes, 3 times a day. In order to prevent cell proliferation, the migration assay was performed with serum free media for only 24 hours. On the next day, non-migrated cells from the upper surface of the membrane were carefully removed by a cotton swab. The cells that migrated to the other side of the membrane were fixed in cold methanol for 10 minutes, stained with 1.4% crystal violet and washed three times with distilled water. Cell migration was evaluated by photomicrographs from five randomly chosen fields (x100) per insert for counting the number of migrated cells and the adhesion area using the Image-Analysis J 1.45S software as described previously [13]. The numbers of migrated cells and cell area per cell between groups were compared.

### RNA analysis by semi-quantitative reverse transcription–polymerase chain reaction (RT-PCR)

To perform semi-quantitative RT-PCR, after rinsing with 1.8% NaCl, 2.9% KCl, 10% NaH_2_PO_4_, 9.7% KH_2_PO_4_ in culture medium for 2 minutes, 3 times a day, total mRNA of hGFs was isolated by using Trizol reagent (#2302700, Prime, Gaithersburg, MD). First-strand cDNA was synthesized using reverse transcriptase reaction by ImProm-II Reverse Transcription System (#A3800, Promega Corporation, Madison, WI) and semi-quantitative PCR was performed following the manufacturer's manual.

PCR primer for type I collagen (COL1), fibronectin (Fn) and Focal Adhesion Kinase (FAK) were used to screen extracellular matrix gene expression (Table 1). All bands were scanned, analyzed, and normalized with the expression of the housekeeping gene glyceraldehyde 3-phosphate dehydrogenase (GAPDH) using Bio-1D software version 15.03 (Vilber Lourmat, Marne La Vallée, France). Three independent experiments were repeated in each sample to compare the fold change of gene expression.

**Table 1:** Primers used in RT-PCR.

Primer	Sequence ID	Sequence (Forward and Reverse 5’-3’)	Base pairs	Cycles
COL1	NM_000088.3	F: GCA AAG AAG GCG GCA AA	500	22
		R: CTC ACC ACg ATC ACC ACT CT		
FAK	NM_004967.3	F: CAA TCC CAC ACA TCT TGC TGA	186	38
		R: AGC CGG CAG TAC CCA TCT ATT		
Fn	NM_002026.2	F: GGA TCA CTT ACG GAG AAA CAG	401	28
		R: GAC ACT AAC CAC ATA CTC CAC		
GAPDH	NM_002046.3	F: TgA Agg TCg gAg TCA ACg gAT	396	22
		R: TCA CAC CCA TgA CgA ACA Tgg		

### Immunofluorescent staining

Immunofluorescent staining was performed to detect the distribution of Focal Adhesion Kinase (FAK) and actin filaments (F-actin). Firstly, hGFs were seeded onto 8-well chamber slides (#154534, Lab-Tek^®^ II Chamber Slide w/Cover, Thermo Fisher Scientific, Rochester, NY) at 5,000 cells/well (7,150 cells/cm^2^). For the next day, the cells were rinsed with 1.8% NaCl for 2 minutes, 3 times a day. After 24h, the medium was removed and the cells were rinsed 2 times with PBS, fixed in 3.7% formaldehyde in PBS for 20 minutes at room temperature and permeabilized in 0.5% Triton X-100 in PBS for 2 minutes. The cells were then incubated with 2μg/ml of anti-FAK, clone 4.47, Alexa Fluor^®^ 488 conjugate (#16–233, Merck Millipore, Darmstadt, Germany) in PBS for 1 hour at room temperature. The cells were rinsed with PBS and incubated for 15 minutes with rhodamine-phalloidin (#R415, Invitrogen, Life Technologies Corporation) (1:500 dilution) for F-actin staining. Cells were washed again and counterstained with 4',6-Diamidino-2-Phenylindole, Dihydrochloride (DAPI, #D1306, Life Technologies Corporation) for nuclear staining. After covering the samples with ProLong^®^ Gold Antifade Reagent (#P36934, Life Technologies Corporation) to protect fluorescent dyes from fading, slides were observed by a fluorescence microscope (Axio Observer.Z1, Carl Zeiss, Oberkochen, Germany).

### Data analysis

All experiments were repeated three to four times. For statistical analysis, independent samples comparison t-test, one-way ANOVA with Dunnett T3 (unequal variances) or Tukey HSD post-hoc test (equal variances) will be used to compare between groups using SPSS v.21 (IBM, New York, NY, USA) with the level of significance being 0.05.

## Results

### Rinsing with NaCl stimulated the migration of hGFs

**Fig 1:**
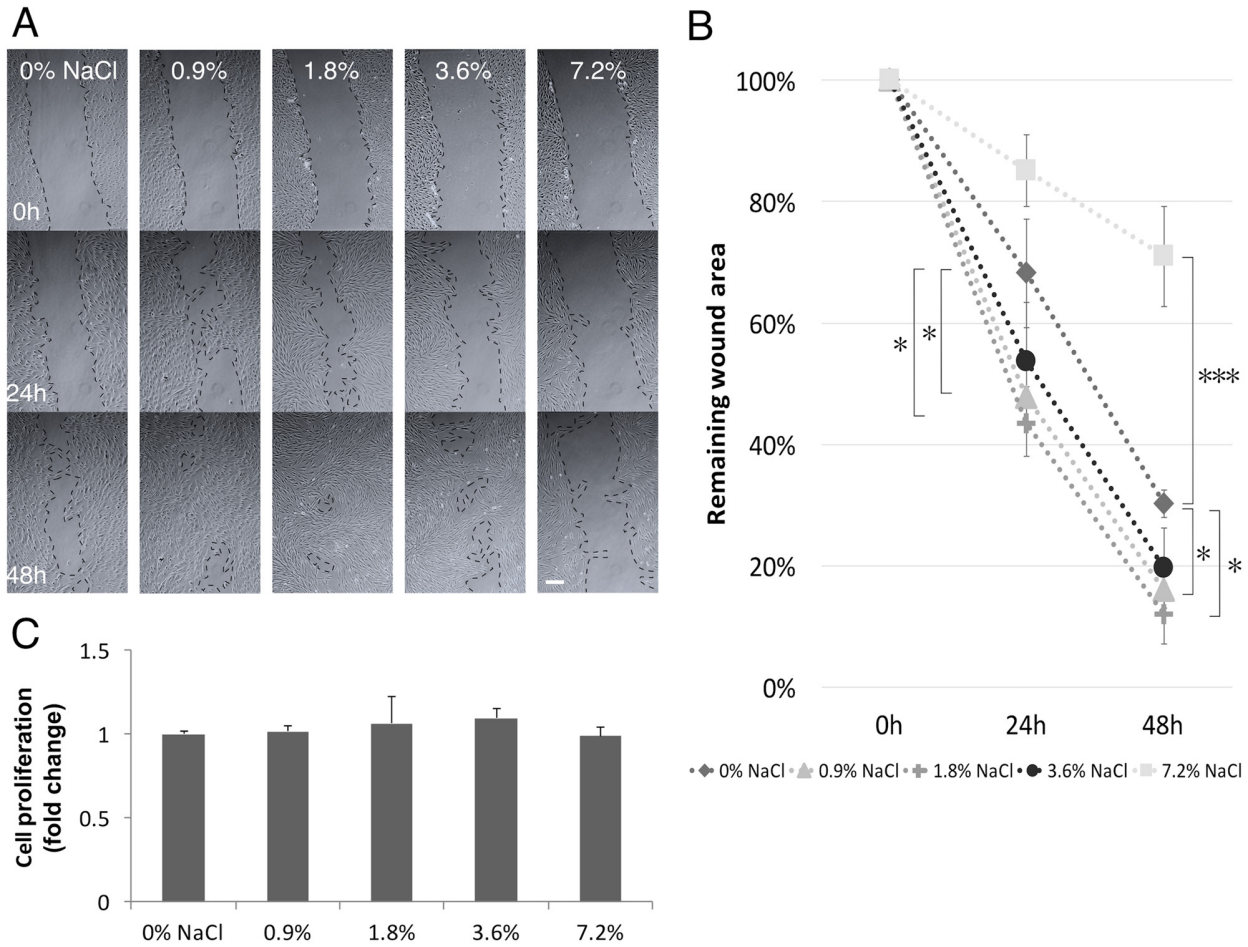
Short-term NaCl rinsing enhanced wound healing in hGFs. A) Scratch-test assay. B) Remaining wound area was normalized with the time point 0 h. C) NaCl rinsing did not alter the proliferation of hGFs by MTT assay. One-way ANOVA vs. 0% NaCl, *p< 0.05, **p<0.01, ***p<0.001, scale bar 100 μm.

In order to test the effect of short-term rinsing with NaCl on “wound healing”, its effect on hGFs was analyzed in a scratch-test assay. Rinsing with 0.9 and 1.8% NaCl solution significantly enhanced “wound closure” compared to control group. The 1.8% NaCl solution had the strongest effect as demonstrated by minimum remaining “wound” area at both 24 and 48 hours. A higher concentration of NaCl at 7.2% gave rise to an opposite result, the cell free area in this group remained rather wide even after 48 hours (Fig 1A and 1B). These results indicated that rinsing with a relatively low concentration of NaCl solution promoted the migration of hGFs in an artificial wound *in vitro*.

### Rinsing with NaCl increased hGF migration and adhesion area but did not affect cell proliferation

**Fig 2:**
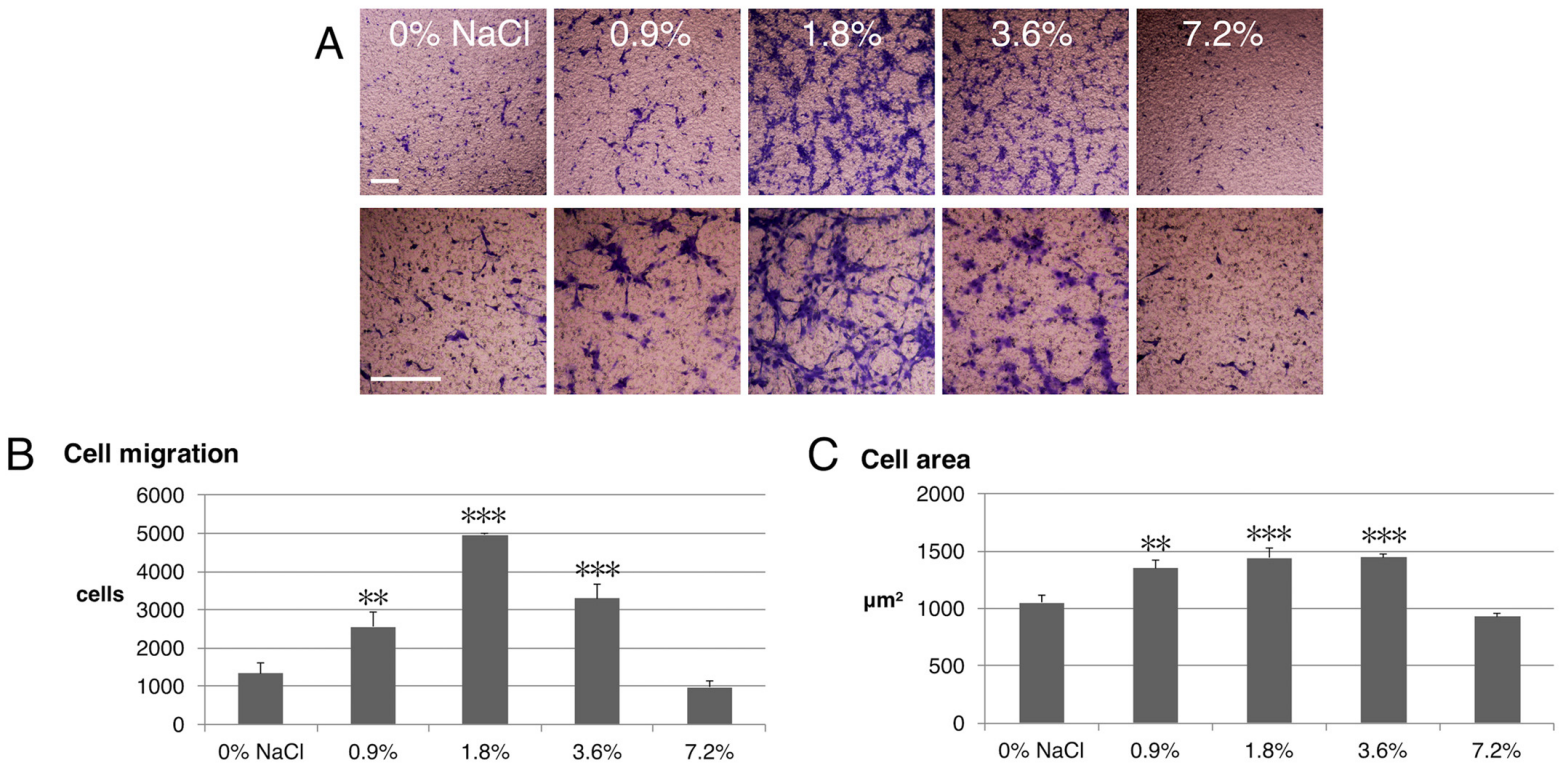
Short-term NaCl rinsing enhanced the migration of hGFs. A) Migrated cells were stained with crystal violet after transwell migration assay. Migrated cells B) and cell area C) were measured and compared by one-way ANOVA vs. 0% NaCl, scale bar 100 μm.

To clarify part of the mechanisms of NaCl rinsing on the behavior of hGFs, cell viability and migration were assessed by MTT and transwell migration assays, respectively. From growth curve analysis, doubling time of GFs were calculated to be 30.4 h. (S1 Fig). Rinsing with NaCl did not exert any effect on hGFs viability within 24 h. at any concentration tested (0–7.2%) (Fig 1C). However, cell migration was altered significantly. Short-term rinsing with NaCl at the concentration of 0.9, 1.8 and 3.6, but not 7.2%, significantly increased hGFs migration through a polycarbonate membrane (Fig 2A and 2B). This data corresponds with the scratch assay in which 1.8% NaCl demonstrated the highest stimulatory effect on cell migration. Treatment with NaCl also increased cell adhesion area, which result in a larger cell area (Fig 2C).

### Rinsing with NaCl did not affect hNOKs

**Fig 3:**
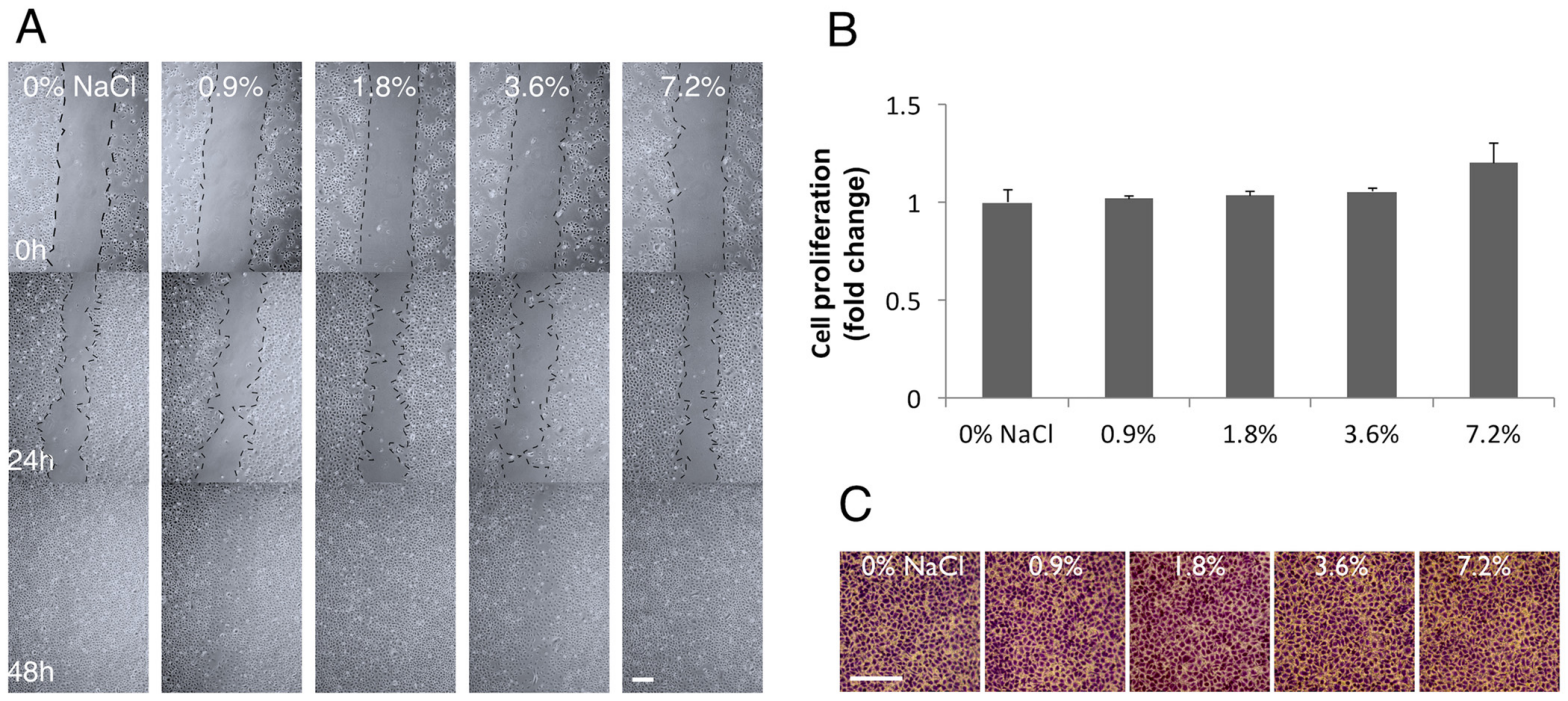
Short-term NaCl rinsing did not affect hNOKs. A) Scratch-test assay. B) Cell proliferation by MTT assay. C) Cell migration by transwell migration assay. One-way ANOVA vs. 0% NaCl, n = 3, scale bar 100 μm.

To investigate whether NaCl rinsing affected migration of human oral keratinocytes, similar experiments were performed using hNOKs. The results clearly demonstrated that rinsing with NaCl did not exhibit any effect on the migration or viability of hNOKs at any concentration tested (0–7.2%) (Fig 3).

### Rinsing with NaCl up-regulated COL1, Fn and FAK expression of hGFs

**Fig 4:**
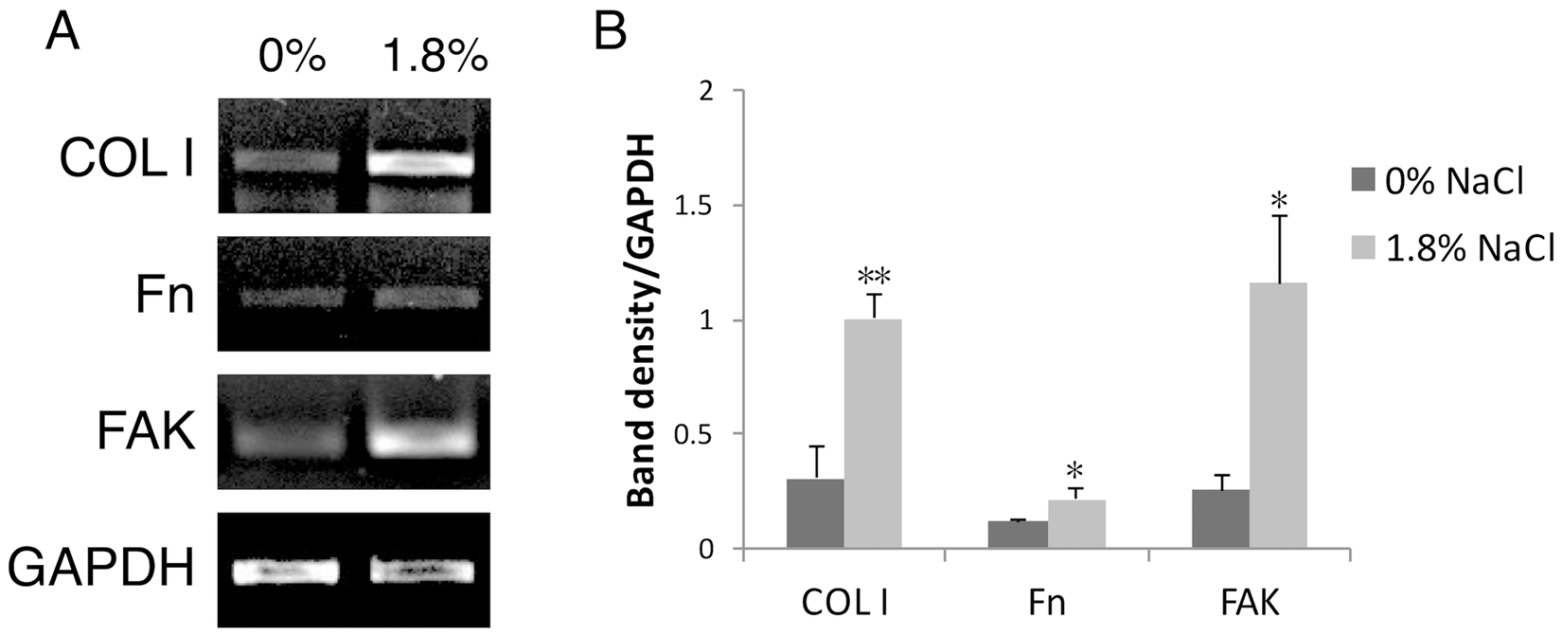
Short-term treatment at 1.8% NaCl altered the expression of COL1, Fn and FAK. A) mRNA expression by RT-PCR. B) Band-density quantitative analysis. Independent t-test 0% vs. 1.8% NaCl.

We further examined whether NaCl altered extracellular matrix production which is a crucial step of wound healing. More than three-fold induction of COL1 mRNA expression was found in the NaCl-rinsed group. A slight but significant up-regulation of Fn mRNA was observed. Remarkably upregulation of FAK mRNA expression was also observed after rinsing with 1.8% NaCl. (Fig 4). This data indicated that rinsing with NaCl up-regulated the expression of extracellular matrix and cytoskeletal related genes in hGFs. RT-PCR also showed that there was no up-regulation of TGF-β, MMP1, MMP2 after NaCl rinsing (S2 Fig).

### Rinsing with NaCl promoted FAK and F-actin re-organization of hGFs

**Fig 5:**
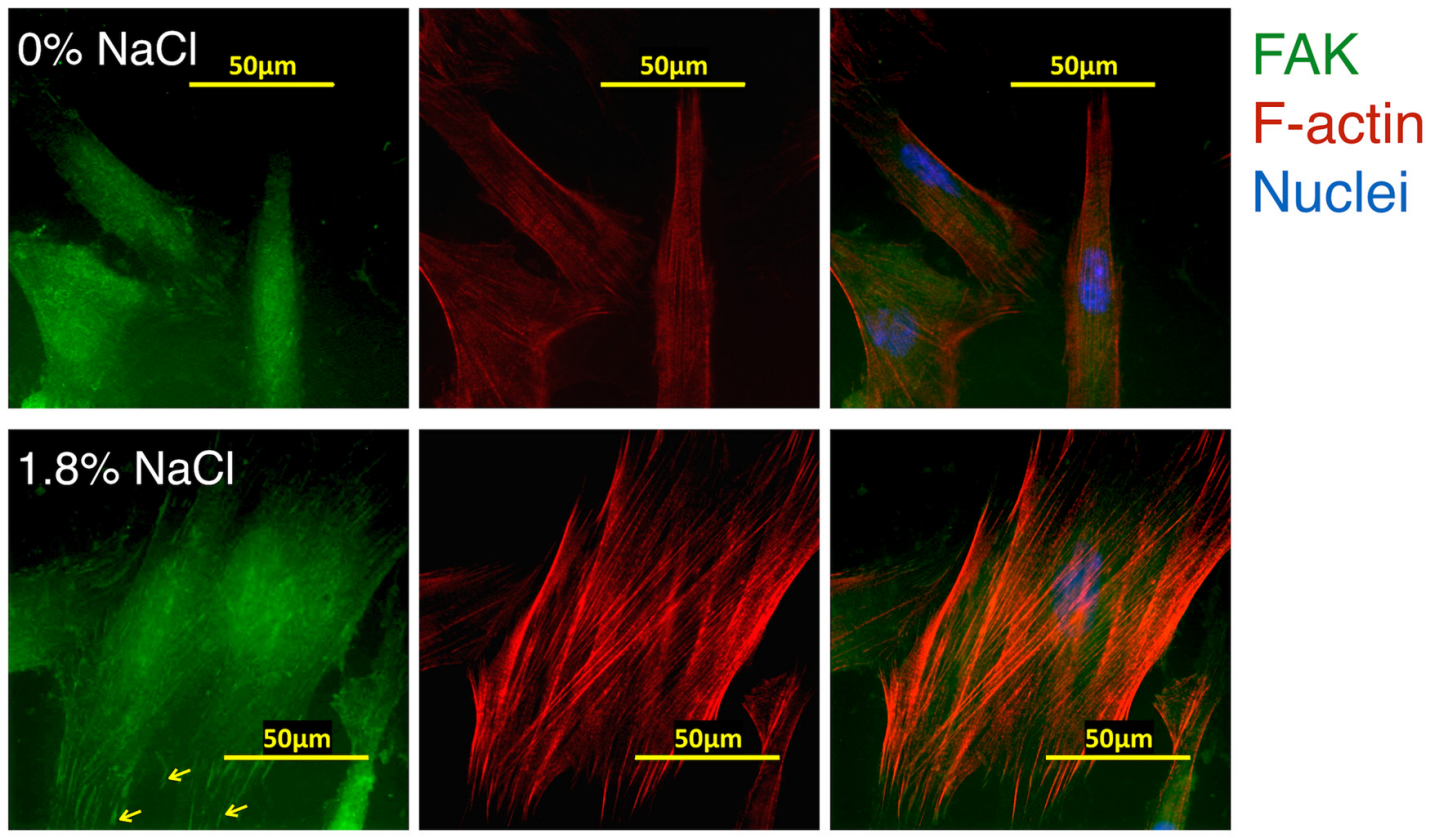
**Short-term treatment at 1.8% promoted the expression of FAK and F-actin** in hGFs by immunofluorescence, (head arrows: peripheral FAK scale bar 50 μm).

Fluorescent staining was used to evaluate the distribution of focal adhesion kinase (FAK) and actin filaments (F-actin) in hGF cultures after rinsing with NaCl. The results indicated a higher expression of cytoskeleton-related proteins after 1.8% NaCl rinsing (Fig 5). FAK was distributed throughout the cells in control group, but in the NaCl-treated cells FAK was located in well-defined focal adhesions sites at the periphery of the fibroblasts. This condition also induced actin rearrangement with led to a marked increase in dense parallel arrays of bundled F-actin fibers. In summary, up-regulation of FAK and F-actin, molecules responsible for cell migration, were observed after rinsing with 1.8% NaCl solution.

### Chloride ion is a key player of NaCl effect on hGFs cell migration

**Fig 6:**
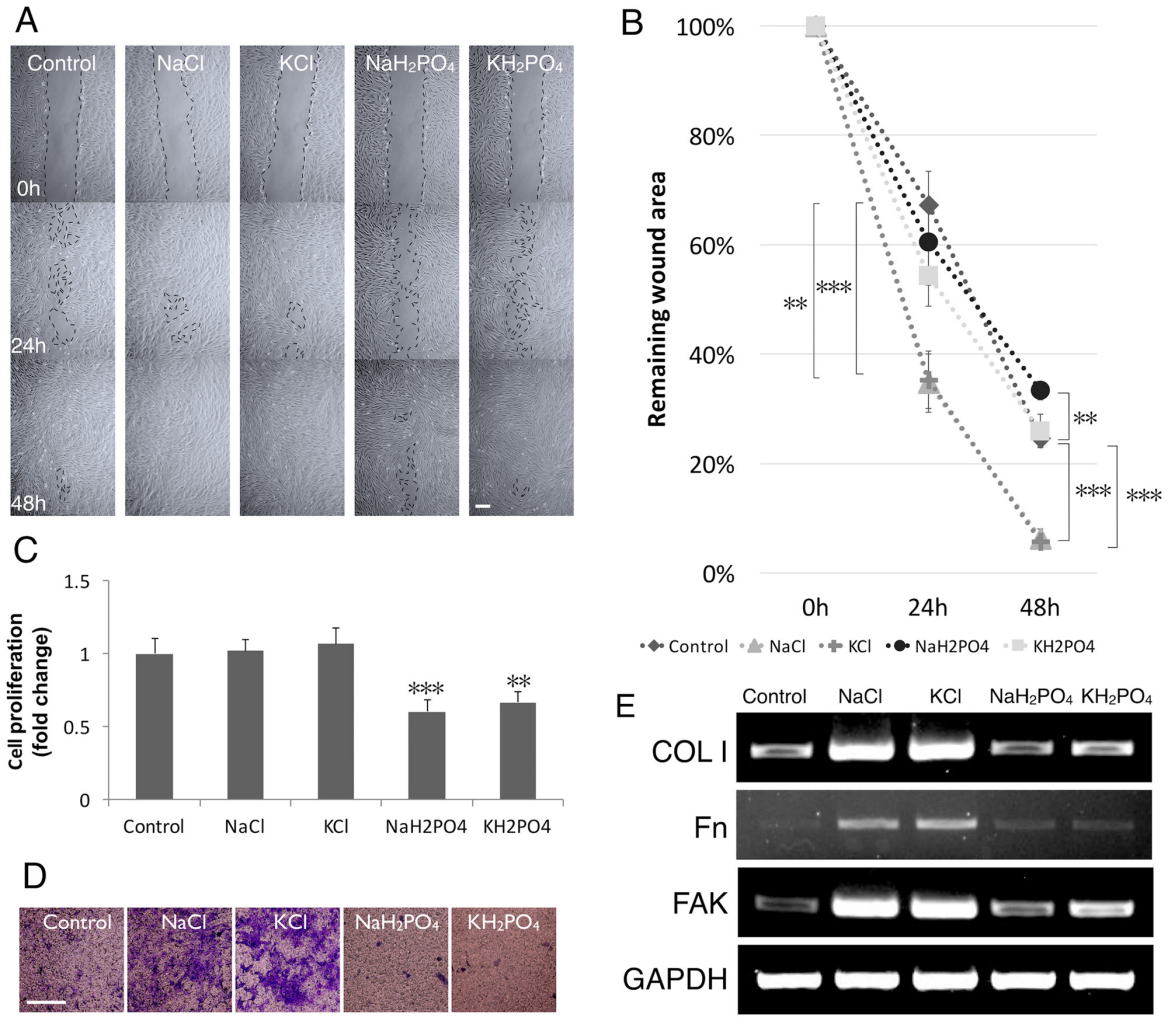
Chloride involved in the migration of hGFs. A) Scratch-test assay. B) Remaining wound area was normalized with the time point 0 h. C) NaCl, KCl rinsing did not alter the proliferation of hGFs by MTT assay while NaH_2_PO_4_, KH_2_PO_4_ did. D) NaCl, KCl rinsing increased migrated cells by transwell migration assay. One-way ANOVA vs. 0% NaCl, scale bar 100 μm. E) Gene expression of COL1, Fn, FAK with and without NaCl, KCl, NaH_2_PO_4_ and KH_2_PO_4_ rinsing by RT-PCR.

To clarify whether the effect of NaCl is caused by sodium or by chloride, the equivalent mole of sodium and chloride was analyzed in more detail. The solutions were: KCl (2.9%), NaH_2_PO_4_ (10%), and KH_2_PO_4_ (9.7%). Scratch-test assay showed that rinsing with KCl had a same effect on “would healing” as NaCl while the sodium and potassium phosphate salts resulted in a reverse effect (Fig 6A and 6B). Chloride salts did not exhibit any effect on hGFs viability. However, NaH_2_PO_4_ and KH_2_PO_4_ decreased the cell number (Fig 6C). In line with the data obtained from the scratch assay, rinsing with NaCl and KCl significantly stimulated cell migration as assessed with the transwell migration assay. This effect was not observed with sodium and potassium phosphate salts (Fig 6D). These results indicate chloride as a key player in promoting hGFs migration. In addition, RT-PCR indicated that NaH_2_PO_4_, KH_2_PO_4_ did not upregulate the mRNA expression of COL1, Fn and FAK while KCl had the same effect as NaCl (Fig 6E). The data indicated a role of Cl^-^ in the cellular response of hGFs.

## Discussion

In the present study, we demonstrated that short-term rinsing with NaCl solution promotes migration which is an important process during wound healing in primary culture of human gingival fibroblast. This data will provide the first scientific evidence supporting the long held belief in the benefits of using NaCl mouth rinse to support healthy gums and hasten oral ulcer healing.

Rinsing the mouth with salt solution is recognized as the most preferable method for oral wound care. It is an economic and nontoxic way to provide a moist environment to facilitate the healing of oral wounds [14, 15]. In our model we tried to mimic the practical habit of performing oral rinse, whereby the cells were rinsed 2 min, 3 times a day. Even with a brief exposure to hypertonic solution, appropriate doses of NaCl (0.9–1.8%) promoted hGFs cell migration and extracellular matrix production. Using too high concentration of NaCl (7.2%) was shown to result in adverse effects. These data, therefore, provide an appropriate recommended dosage of NaCl for oral rinse. One should consider carefully the dosage of NaCl since a high NaCl concentration especially with long-term exposure proved to cause DNA damage and inhibit its repair. This may lead to apoptosis or may affect cellular survival and their function both in cell culture and *in vivo* [16, 17]. However, prolonged exposure to hypertonicity of certain cell types can result in adaptation to osmotic stress. For example, incubation of cultured fibroblasts and myoblasts in medium containing NaCl for 24h resulted in an increased cell diameter and adhesion area [8]. These findings suggest that the cells over-compensate volumetric changes by increasing cell volume and adhesion area under hyperosmotic conditions; a phenomenon also found in our study. Growth curve analysis demonstrated that doubling time of hGFs was 30.4 h (S1 Fig). The log phase was starting from day 1 to day 8. Previous studies showed that doubling time of GFs was 33.6 or 33.9 h [18, 19]. Though, our model of wound healing assay (48h) was conducted within the log phase of growth curve, we do not found the difference in cell viability between groups at any time point as demonstrated by the data of MTT assay at 24 and 48 h. However, the transwell migration assay did demonstrated the significant difference between NaCl-rinsed groups and the control. Hence, we concluded that NaCl enhance wound healing by affect cell migration but not proliferation.

NaCl did not have any effect on migration and/or viability of hNOK cells. Previous studies showed that in the presence of EGF human epidermal keratinocytes expressed strong in vitro wound epithelialization capacity [20]. Although the keratinocyte is one of the key players in wound healing after tissue injury [21], it appears not to respond to NaCl in a way comparable to that of the fibroblasts. Further studies are needed to clarify the role of this cell type in wound healing in other aspects.

In a previous study it was shown that hypertonicity induces rapid F-actin polymerization [16]. In CHO fibroblast, hypertonicity induces actin skeleton remodeling and de novo F-actin assembly at the cell periphery [22]. The same phenomenon was also observed in our study. Moreover, extracellular matrix such as collagen and fibronectin play an essential role in wound healing via the promotion of cell migration, especially in GFs [9]. The roles of FAK in cell migration and cytoskeletal changes upon hyperosmotic stress or NaCl treatment reported previously are in line with our data [23, 24]. We found also that NaCl up-regulated and re-localized FAK into well-defined spots at focal adhesions Concomitant with an increased spreading of hGFs, rinsing with NaCl solution, especially at 1.8%, promoted FAK up-regulation and re-localization. In conjunction herewith, we found an up-regulation of COL1 and Fn but not TGF-β, MMP1, MMP2 (S2 Fig). These findings strongly support the potential effect of NaCl to promote gingival wound healing. The effect of NaCl on upregulation of ECM components promote a scar formation is under question. TGF-β, MMP1, MMP2 which are involved in cell growth, cell proliferation, cell differentiation and matrix degradation. Previous study showed that stimulation of urokinase production by exogenous TGF- β1 is involved in the responses of activated gingival granulation-tissue fibroblasts to tissue injury [25]. Our study reveals that rinsing with NaCl did not alter TGF-β expression in gingival wound created in vitro. Further investigation need to clarify other molecules.

KCl, NaH_2_PO_4_, KH_2_PO_4_ were used to obtain insight in the nature of the ion, sodium and/or chloride, responsible for the increased migration. Rising with either NaCl or KCl promoted hGF cell migration while sodium, potassium and phosphate salts had no effect. Moreover, NaH_2_PO_4_, KH_2_PO_4_ did not upregulate the mRNA expression of COL1, Fn and FAK while KCl showed the same results as found with NaCl. We, therefore, conclude that the ion responsible for the stimulation of migration is chloride. Several studies indicated the role of chloride channels and transporters in cell volume and migration [26]. In glioma cells, the permeation of chloride ions through chloride channels is obligatory for cell migration while volume-activated Cl channels contribute to the cell-cycle-dependent regulation of HeLa cell migration [27, 28]. Whether hGFs express such channels and, if so, whether these play a role in the migration of these cells, is unknown and needs further investigation.

There are several studies that tried to detect the ability of commercial mouth-rinses to promote wound healing of hGFs. These studies had, however, contradictory results. For example, chlorhexidine can significantly reduce both collagen and non-collagen protein production of hGFs *in vitro*, and alcohol-containing mouthrinses inhibited hGFs adhesion and viability while essential oil mouthwashes displayed no detectable detrimental effects on human gingival and PDL fibroblasts [29–31]. There is a clinical study indicating the effectiveness of saline rinses to treat mucositis in patients with oral irradiation [32]. Although our study is based on an *in vitro* model, it provides insight into the mechanisms by which NaCl influences the behavior of hGFs. To confirm the advantage of NaCl rinsing clinical studies are needed.

In our model, hGFs were cultured in growth medium which already contains a small amount of NaCl (about 0.4%) resembling the isotonic solution. The cells were therefore exposed to slightly higher dosages of NaCl than indicated in the experiment. Given that 1.8% NaCl was the most effective concentration, it should be re-calculated to an actual amount of 2–2.2% NaCl. Extrapolating from this concentration, we would suggest to mix approximately one teaspoon full (5 g) of NaCl in a cup of water (250 ml) to use as oral rinse.

In conclusion, NaCl solution stimulates hGF cell migration, alters the organization of cytoskeletal molecules (FAK and F-actin), and enhances extracellular matrix gene expression (COL1 and Fn). These data provide the first scientific evidence to support the application of salt solution as mouth-rinse in conjunction with routine oral care to promote oral wound healing.

## Supporting Information

S1 Fig Proliferation and growth curve of GFs. A) MTT assay of GFs after 24h. B) Growth curve of GFs in 12 days by 0.4% trypan blue (± SD, n = 3, Student's t-test). C) Doubling time of GFs.
(TIF)

S2 Fig Gene expression of TGF-β, MMP1, MMP2 with and without NaCl rinsing. A) mRNA bands by RT-PCR. B) Primers for RT-PCR.
(TIF)
