# Supplementary figures and images for: Rinsing with Saline Promotes Human Gingival Fibroblast Wound Healing In Vitro
Source: PLoS One. 2016 Jul 21;11(7):e0159843. doi: 10.1371/journal.pone.0159843 (PMC4956236; doi:10.1371/journal.pone.0159843)

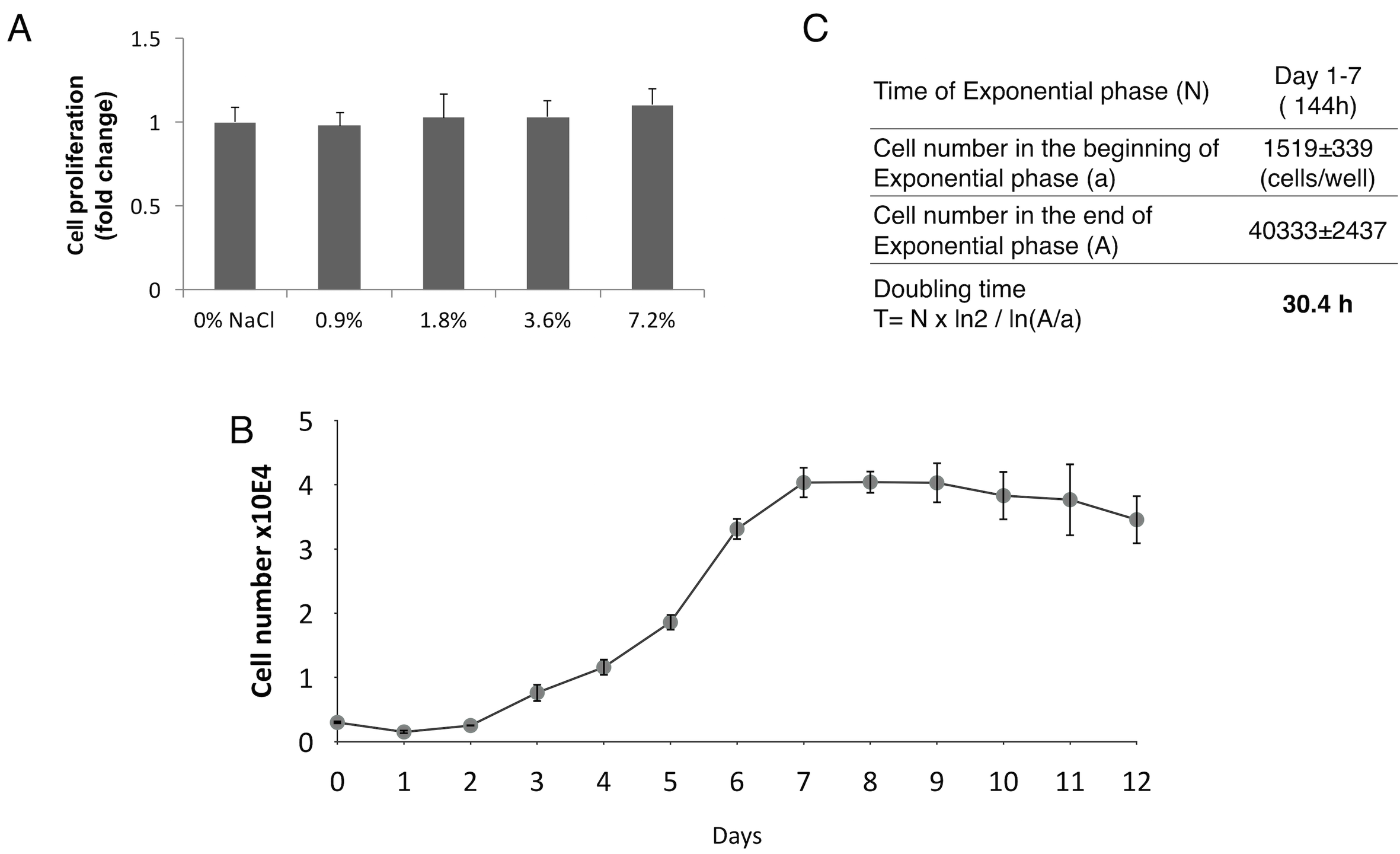

Supplement: S1 Fig — A) MTT assay of GFs after 24h. B) Growth curve of GFs in 12 days by 0.4% trypan blue (± SD, n = 3, Student's t-test). C) Doubling time of GFs. (TIF) [file pone.0159843.s001.tif]

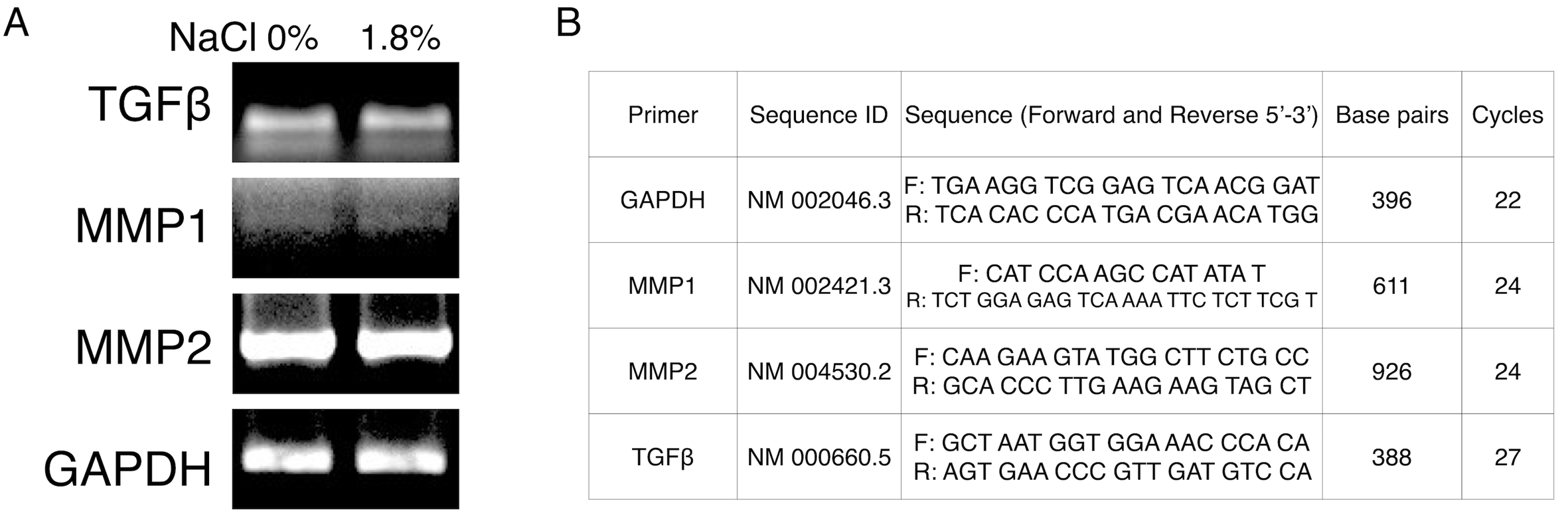

Supplement: S2 Fig — A) mRNA bands by RT-PCR. B) Primers for RT-PCR. (TIF) [file pone.0159843.s002.tif]
